# Growth-Promoting Characteristics of Fungal and Bacterial Endophytes Isolated from a Drought-Tolerant Mint Species *Endostemon obtusifolius* (E. Mey. ex Benth.) N. E. Br

**DOI:** 10.3390/plants12030638

**Published:** 2023-02-01

**Authors:** Abdulazeez A. Ogbe, Shubhpriya Gupta, Wendy A. Stirk, Jeffrey F. Finnie, Johannes Van Staden

**Affiliations:** 1 Research Centre for Plant Growth and Development, School of Life Sciences, University of KwaZulu-Natal Pietermaritzburg, Private Bag X01, Scottsville 3209, South Africa; 2 Department of Botany, Lagos State University, Km 15, Badagry Expressway, Lasu Post Office, Ojo, P.O. Box 0001, Lagos 102101, Nigeria; 3 Laboratory of Growth Regulators, Faculty of Science, Palacký University & Institute of Experimental Botany AS CR, v.v.i, Šlechtitelů 11, 78371 Olomouc, Czech Republic

**Keywords:** abiotic stress, Lamiaceae, medicinal plant, microbes, secondary metabolite

## Abstract

Endophytes are primarily endosymbiotic bacteria and fungi that colonize the interior tissues of their host plant. They enhance the host plant’s growth and attenuate adverse effects of biological stress. Endophytic species of many indigenous plants are an untapped resource of plant growth-promoting microorganisms that can mitigate abiotic stress effects. Thus, this study aimed to isolate endophytes from the roots and leaves of the medicinal plant *Endostemon obtusifolius* to evaluate their in vitro growth-promoting capacities and drought tolerance and to characterize the most promising species. Twenty-six endophytes (fourteen bacteria and twelve fungi) were isolated and cultured from the roots and leaves of *E. obtusifolius*. All 26 endophytes produced flavonoids, and 14 strains produced phenolic compounds. Of the 11 strains that displayed good free radical scavenging capability (low IC_50_) in the 1-1-diphenyl-1-picryhydrazyl radical scavenging assay, only three strains could not survive the highest drought stress treatment (40% polyethylene glycol). These 11 strains were all positive for ammonia and siderophore production and only one strain failed to produce hydrogen cyanide and solubilize phosphate. Seven isolates showed aminocyclopropane-1-carboxylate deaminase activity and differentially synthesized indole-3-acetic acid. Using molecular tools, two promising symbiotic, drought stress tolerant, and plant growth-enhancing endophytic species (EORB-2 and EOLF-5) were identified as *Paenibacillus polymyxa* and *Fusarium oxysporum*. The results of this study demonstrate that *P. polymyxa* and *F. oxysporum* should be further investigated for their drought stress mitigation and plant growth enhancement effects as they have the potential to be developed for use in sustainable agricultural practices.

## 1. Introduction

Fluxes in environmental parameters predispose plant species to different types of abiotic stress, such as water deficit, salinity, heavy metal toxicity, high and low-temperature stress, UV radiation stress, and nutrient deficiency [1]. Generally, abiotic stress is detrimental to medicinal plants, influencing growth, development, and related metabolic pathways that are responsible for synthesizing valuable phytochemicals [2]. The consequences of drought stress are predicted to be more devastating in the coming decades [3]. Water scarcity is the most common and significant environmental issue affecting many regions of the world and is directly linked to other stress types, including salinity and heat stress [4]. In plants, severe drought stress causes water loss and stomatal closure, limits gaseous exchange, disrupts nutrient uptake, impairs metabolic activities and cell division, stimulates reactive oxygen species accumulation in cells, and can lead to cell damage or death [5]. However, depending on the severity of the drought stress, exposure period, genetic makeup, and growth stage of the plant, certain plants tolerate or survive drought due to a series of biochemical and physiological processes mediated by their mutualistic association with microbial endophytes [6]. Plant–endophyte interactions mainly promote plant growth and health [7], consequently improving the plant’s resistance abilities to combat environmental stress [8].

One ubiquitous feature of plants is the harmless endophytes within their living tissues [9]. These are primarily endosymbiotic bacteria and fungi that colonize the interior tissues of a plant. This symbiotic relationship is flexible and may be beneficial to the host plant while being pathogenic to other plant species. This is influenced by the endophyte species, environmental conditions, and the host plant’s physiological state [10]. Some medicinal plant-associated endophytes have the ability to synthesize a diverse range of natural products that are useful in promoting plant growth under moderate or stressful environmental conditions [11,12]. Endophytes stimulate plant growth, development, and productivity by solubilizing inorganic phosphates [13], decreasing ethylene production in the plants [14], and fixing inert atmospheric nitrogen [13]. The beneficial impact of endophytes may also be through the synthesis of hydrolytic enzymes and plant growth regulators such as auxins [15], ammonia [16], and siderophores [17]. Siderophores are microbial iron-scavenging low-molecular secondary metabolites that ensure sufficient iron supply to the microbes and their host during iron shortages [18]. Endophytes may be cultured in vitro after a series of sterilization procedures [19].

Globally, plant species in the Lamiaceae (mint) family are the most explored indigenous plants owing to their use as herbs and spices [20], memory boosters, and antioxidants [21]. *Endostemon obtusifolius* (E. Mey. ex Benth.) N.E.Br. is a relatively unknown and under-explored member of the mint family that grows in semi-arid regions of southern Africa. Its pharmacological activities have not been verified, although it has free radical-scavenging and acetylcholinesterase-inhibiting properties [22]. The endophytic composition of its organs, its drought tolerance capability, and the plant growth-promoting properties of the endophytes have not been investigated. Therefore, the aim of this study was to isolate the bacterial and fungal endophytes in the leaves and roots of *E. obtusifolius* and to investigate the in vitro plant growth-promoting properties and drought tolerance of these endophytic species.

## 2. Results

### 2.1. Sterilization, Isolation, and Purification of Endophytes

**Figure 1:**
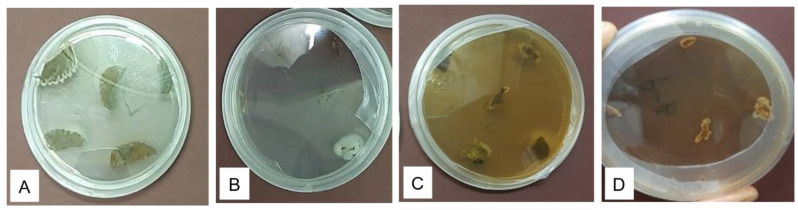
Growth of (**A**,**B**) endophytic bacteria and (**C**,**D**) fungi emerging from the leaves and roots of *Endostemon obtusifolius*.

**Figure 2:**
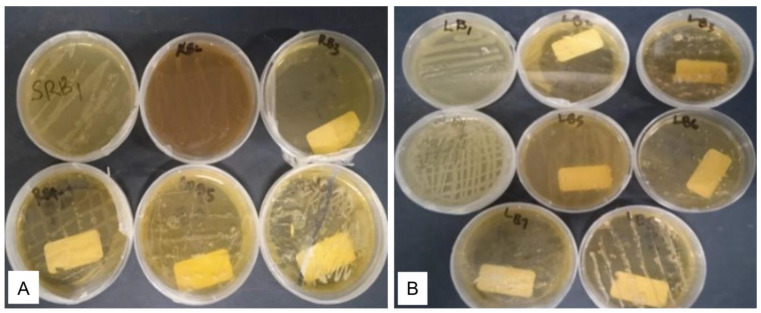
Pure culture plates of bacteria isolated from (**A**) the roots and (**B**) leaves of *Endostemon obtusifolius*.

**Figure 3:**
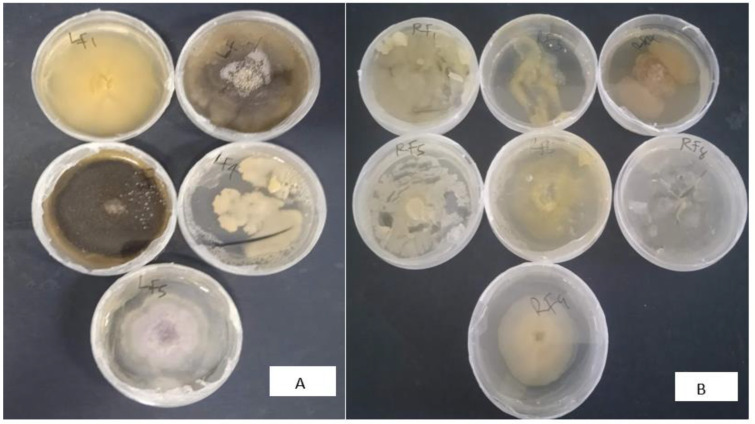
Pure culture plates of fungi isolated from (**A**) the leaves and (**B**) roots of *Endostemon obtusifolius*.

The endophytic microbial community of the healthy roots and leaves of *E. obtusifolius* were evaluated using surface-sterilized explants cultivated on potato dextrose agar (PDA) and nutrient agar (NA; Figure 1). The two sterilization check methods, namely culturing aliquots of water from the last explants rinsed onto culture media and imprinting the sterilized explant surfaces onto nutrient media, were effective as no microbial growth was observed on the control culture plates. Thus, any isolates obtained from the plant material were considered endophytes. Colonies with different morphologies were selected and sub-cultured on newly prepared media, and 26 pure cultures were obtained. Six endophytic bacteria (designated as EORB-1 to EORB-6) and seven endophytic fungi (designated as EORF-1 to EORF-9) were obtained from the roots, and eight endophytic bacteria (designated as EOLF-1 to EOLF-9) and five endophytic fungi (designated as EOLF-1 to EOLF-5) were isolated from the leaves (Figure 2 and Figure 3).

### 2.2. Total Phenolic and Flavonoid Content Estimation

The total phenolics and flavonoids in the crude extracts of the endophytic isolates differed considerably. The total phenolic content of the extracts, expressed in gallic acid equivalents (GAE), ranged from 0.27 to 9.80 mg GAE/mg DW of extracts. The EOLF-5 extract had the highest total phenolic content (expressed in catechin equivalents (CE)), followed by EORF-1 and EORB-2 (Table 1). The fungal isolate EORF-1 had the highest flavonoid content, and EOLB-5 had the lowest (Table 1).

### 2.3. DPPH Radical Scavenging Abilities

Extracts of EORF-1, EOLF-5, and EORB-2 had the highest scavenging power against 1-1-diphenyl-1-picryhydrazyl (DPPH) radicals at 100 µg/mL but were not significantly different from the butylated hydroxytoluene (BHT) control (*p* < 0.05; Table 1). The IC_50_ (the concentration of sample required to scavenge 50% of free radicals) values of the endophytes crude extracts varied widely, with EORF-1 having the lowest IC_50_ values. This was not significantly different from the BHT control (*p* < 0.05; Table 1). Eleven endophytic isolates, namely EORF-1, EORF-5, EORF-8, EOLF-1, EOLF-3, EOLF-5, EORB-1, EORB-2, EOLB-1, EOLB-3, and EOLB-4, with low IC_50_ values and appreciable quantities of flavonoids and total phenolics, were further investigated for their drought tolerance and plant growth-promoting capacities.

### 2.4. The Water-Deficit Resistance Potential of Selected Endophytes

**Figure 4:**
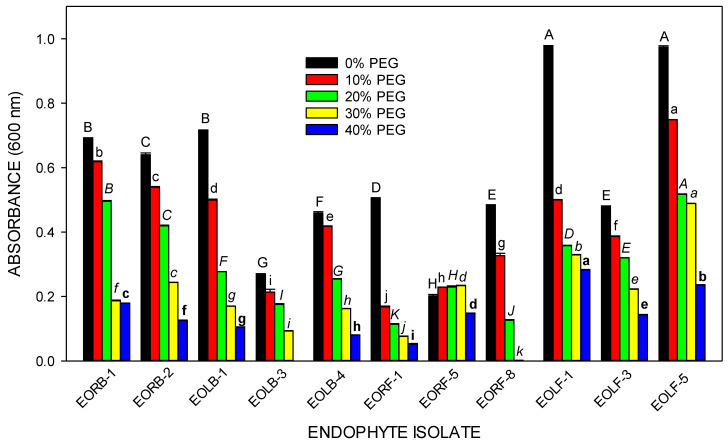
The growth of selected endophytic isolates under non-stressed (0%) and water-deficit conditions treated with increasing (10–40%) PEG 6000 concentrations. Data are mean ± standard error of means (n = 3). Different letters within each PEG treatment show significant differences (*p* < 0.05) between the isolates.

The eleven selected endophytic isolates resisted water-deficit stress initiated by the addition of 10–40% polyethylene glycol (PEG) 6000 to broth media to varying degrees. A general decline in the optical density (OD) of all the endophytic isolates was observed as the concentration of PEG 6000 increased from 10% to 40%. At the highest concentration (40%) of PEG 6000, EORB-1, EORB-2, EORF-5, EOLF-1, and EOLF-5 exhibited significantly high OD values, indicating microbial cell multiplication and drought stress tolerance potency. There was a complete disruption in the growth of EORF-8 and EOLB-3 under the same conditions (Figure 4).

### 2.5. Plant Growth-Promoting Characteristics of Selected Endophytic Isolates

**Figure 5:**
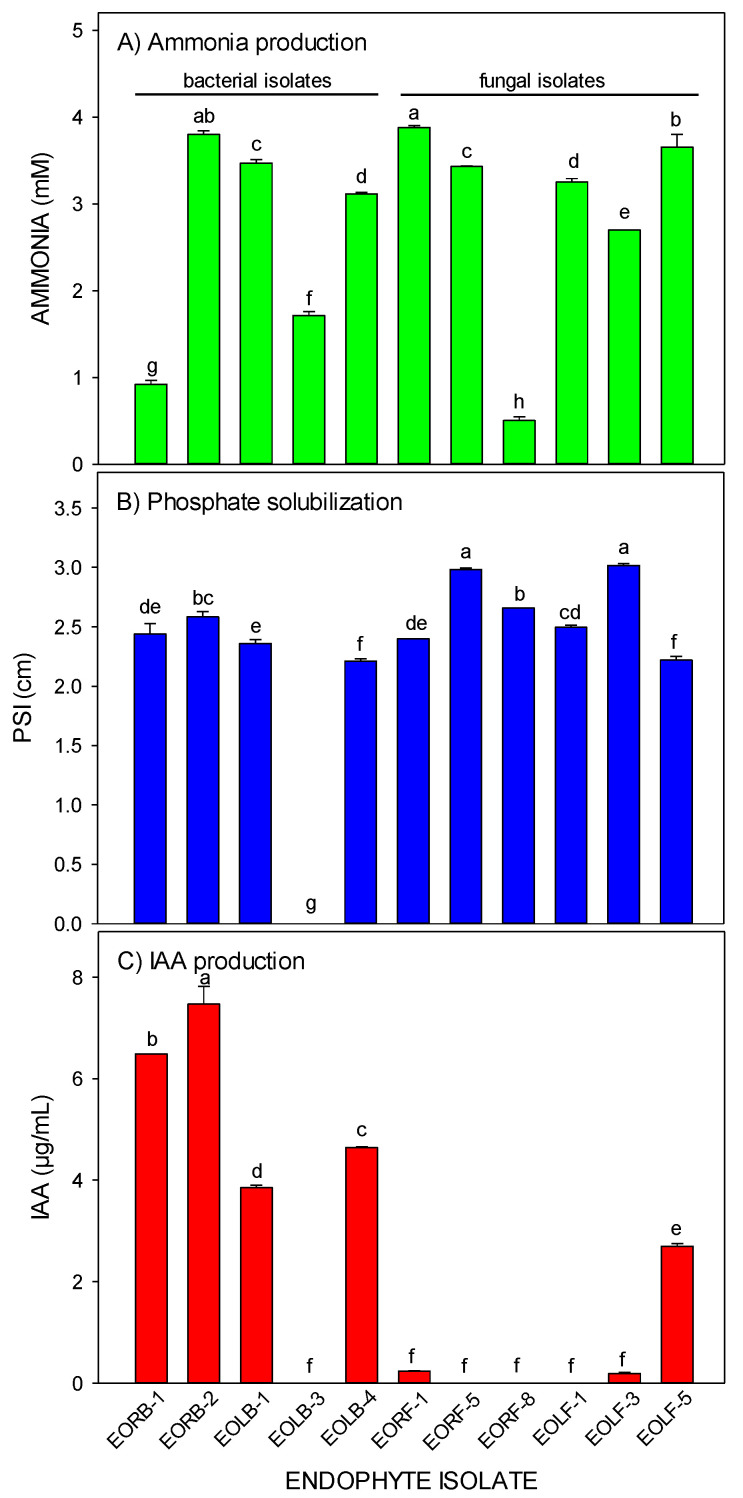
(**A**) Phosphate solubilization index, (**B**) ammonia production, and (**C**) IAA production in selected endophytic isolates. Data are mean ± standard error of means (n = 3). The means with similar letter(s) show non-significant results at *p* < 0.05.

Isolates EORB-2, EOLB-1, EOLB-4, EORF-1, and EOLF-5 showed the most vigorous color intensity, indicating ammonia production. The weakest color intensity occurred in isolates EOLB-3, EORB-1, and EORF-8 (Table 2). The quantity of ammonia produced by the isolates ranged from 0.51 to 3.88 mM, with EORF-1 having the highest ammonia-producing capacity (Figure 5A).

A siderophore-producing capacity occurred in all the isolates, albeit to variable extents, as indicated by the formation of orange halo zones around the colonies on Chrome Azurol S (CAS) agar plates (Table 2). EORB-2, EOLB-1, EOLF-1, and EOLF-5 exhibited the capacity to produce maximum siderophores, with EOLF-5 producing the largest halo zone around its colonies.

All the selected endophytic isolates, except EOLB-3, tested positive for hydrogen cyanide (HCN) production (Table 2). The picric acid pre-soaked filter papers placed underneath the incubated Petri-dish lids of the isolates changed from yellow to deep red or orange, indicating the ability of the isolates to produce HCN. EORB-2, EOLF-1, EOLF-3, and EOLF-5 were the highest producers (Table 2).

Ten of the selected endophytic isolates solubilized tricalcium phosphate on Pikovskaya’s agar (PVK) plates. These isolates produced halo zones around their colonies, indicating their phosphate solubilization potency (Table 2). Phosphate-solubilizing index (PSI) values ranged from 0.00 to 3.02 cm (Figure 5B). The PSI value for isolate EOLF-3 was significantly higher than the PSI values of the other isolates.

The production of indole-3-acetic acid (IAA) was detected in seven (four bacterial and three fungi isolates) of the eleven selected endophytic isolates (Table 2). Isolates EORB-1, EORB-2, EOLB-1, EOLB-4, EORF-1, EOLF-3, and EOLF-5 developed a pinkish color with the addition of Salkowski reagent. EORB-2 synthesized the highest quantity of IAA, followed by EORB-1, EOLB-4, EOLB-1, and the fungal isolates (EOLF-5, EORF-1, and EOLF-3) produced significantly lower amounts of IAA (Figure 5C).

Two of the six fungal isolates (EOLF-3 and EOLF-5) and all of the bacterial isolates utilized aminocyclopropane-1-carboxylate (ACC) as the exclusive nitrogen source. The ability of these isolates to grow on Dworkin and Foster (DF) minimal salt media supplemented with ACC confirmed their aminocyclopropane-1-carboxylate deaminase (ACCD) activity (Table 2).

### 2.6. Molecular Identification of Endophytes

Following the molecular characterization of EORB-2 and EOLF-5, the bacterial isolate (EORB-2) was identified as *Paenibacillus polymyxa* (MT163461.1) and the fungal endophyte (EOLF-5) was a close homolog of *Fusarium oxysporum* (MT560381.1). The BLAST results further revealed 100% and 99.6% identities of the bacterial and fungal endophytes with the rRNA sequences of the related species (Table 3). The assigned GenBank accession numbers of the submitted sequence data are presented in Table 3.

### 2.7. Endophytes Antagonistic Check Using Dual Culture Method

**Figure 6:**
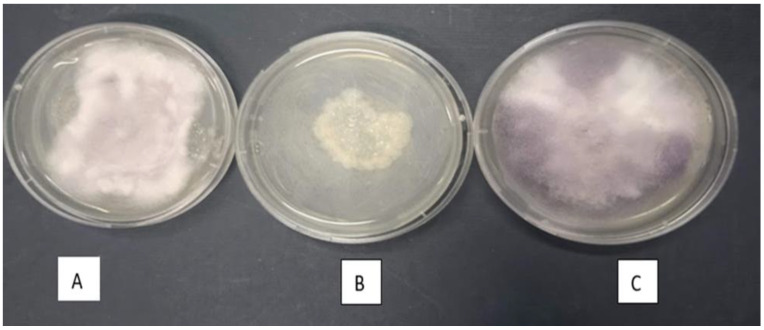
In vitro compatibility test of EORB-2 and EOLF-5. Dual culture plate of (**A**) EORB-2 and EOLF-5 on PDA, (**B**) EORB-2 on PDA, and (**C**) EOLF-5 on PDA.

The isolates *P. polymyxa* (EORB-2) and *F. oxysporum* (EOLF-5) appeared to be compatible symbionts as they grew in vitro with minimal antagonism (Figure 6).

## 3. Discussion

A total of 14 bacterial and 12 fungal endophytes were isolated from the roots and leaves of the medicinal plant *E. obtusifolius*. This was similar to other medicinal plants. For example, 7 bacterial and 5 fungal endophytic species were isolated from the leaves of *Teucrium polium* [23], whereas 127 bacterial and 119 fungal endophytic strains were isolated from the roots, leaves, stems, and fruits of *Schisandra sphenanthera* [24]. The population of endophytes within the host depends on the host species, age, habitat and physiological status, the kind of plant tissues, sampling season, inoculum density, and prevailing environmental conditions [25].

In the present study, the total phenolic and flavonoid contents of ethyl acetate extracts of the isolated endophytic species varied significantly (Table 1). The phenolics and flavonoids detected in the extracts of endophytic species were directly linked to the free radical scavenging abilities of the microbes [26] as was the case in the present study, where the endophytic species extracts with reasonable quantities of total phenolics and flavonoids showed good DPPH free radical scavenging activities (Table 1). Similarly, the methanolic extracts of the endophyte *Eupenicillium senticosum* had high phenolic and flavonoid contents and demonstrated promising in vitro antioxidant properties [27]. The antioxidant capacities of the endophytic fungal species isolated from *Eugenia jambolana* were directly proportional to their total phenolic content [28].

All the endophytes investigated in the present study resisted water stress at 20% PEG, with 82% of the endophytes exhibiting tolerance at 40% PEG (Figure 2). Similarly, all 62 bacteria isolated from *Lepidium perfoliatum* [29], and 16 fungi endophytes isolated from *Triticum aestivum* [30] grew in media culture containing 20% PEG. However, unlike the fungi isolated from *E. obtisufolius*, the fungi isolated from wheat could not survive at a 40% PEG concentration [30]. The scarcity of water usually influences the growth pattern [31], function [32], and productivity [33] of microorganisms. Many plant species that are adapted to grow in dry or semi-arid conditions, such as *E. obtusifolius,* establish symbiotic relationships with drought-tolerant endophytes, which can survive under limited water availability and might benefit their host plant under drought conditions [34].

Plant–endophyte interactions confer abiotic and biotic stress resistance through the production of siderophore, ammonia, HCN, volatile compounds, hydrolytic enzymes, and plant growth regulators [35]. All isolates in this study produced ammonia (Figure 5A, Table 2). The ammonia production capability of endophytes confers resistance in plants against pathogens and directly promotes plant growth through a continuous nitrogen supply [23]. All eleven investigated isolates tested positive for siderophore production in the present study (Table 2). In comparison, 75% of the bacterial isolates from cultivated ginseng produced siderophores [36]. Ten of the selected isolates in the present study showed HCN activity (Table 2), suggesting their possible role as biocontrol agents to inhibit phytopathogens and weed proliferation [18].

Ten isolates investigated in the present study produced clear halo zones around their colonies, indicating their phosphate-solubilizing capacity (Figure 5C). Phosphate solubilization and transportation within plants are essential traits of plant growth-promoting microorganisms. Inorganic phosphate-solubilizing microbes synthesize various enzymes and organic acids, release protons during ammonia assimilation, reduce pH, and chelate cations to release organic and soluble phosphorus to plants [37].

In this current study, EORB-2 and EOLF-5 produced the highest IAA among the bacterial and fungal isolates, respectively, suggesting their plant growth-promoting abilities. IAA is a naturally occurring auxin and a regulator of several developmental processes in plants, including tropisms, organogenesis, cell expansion, division and differentiation, root and pigment formation, mineral nutrition, and plant responses to stress [38]. The stimulation of growth by IAA-producing endophytes relies on the concentration of the IAA produced by the endophyte and the genetic makeup of both the plant and the endophyte involved [39].

In the present study, seven of the eleven investigated endophytes had ACCD activity (Table 2). Many endophytic bacteria [40] and a few endophytic fungi [41] species possess the ACCD gene, which enables them to split the ethylene precursor ACC into ammonia and α-ketobutyrate, thereby improving the fitness of host plants to salinity, drought, heavy metals, and pathogenic attack [42]. Through their phytohormone regulation, osmolyte accumulation, synthesis of ACCD, antioxidants, and several biomolecules (exopolysaccharides), endophytic species tolerate changes in the osmotic potential of their immediate environments [14,43]. Like other stressors, drought enhances the production of the stress hormone ethylene in plants [44]. The inoculation of drought-tolerant and ACCD-producing endophytic species into drought-stress-susceptible plant species has improved the tolerance of stressed plants by reducing the ethylene concentrations within the cells [43,45].

The bacterial and fungal Isolates that exhibited the best plant growth-promoting traits in the present study were identified as *P. polymyxa* (EORB-2) and *F. oxysporum* (EOLF-5). Many studies have reported the isolation and characterization of bacterial and fungal endophytes from plants in the Mint (Lamiaceae) family. Specifically, *F. oxysporum* endophytic strains have been isolated and identified from *Monarda citriodora* [46], *Leucas aspera*, and *Ocimum sanctum* [47]. In contrast, *P. polymyxa,* to the best of our knowledge, has not been isolated from the Mint family but has been obtained from other medicinal plants, including *Lonicera japonica* [48], *Lilium lancifolium* [49], *Ephedra foliate* [50], and *Panax ginseng* [51]. As far as we know, this is the first report on the isolation, characterization, and in vitro plant growth-promoting activity of *F. oxysporum* and *P. polymyxa* obtained from *E. obtusifolius*.

There are inter-species relationships between endophytes in planta [52]. These interactions are governed by competition for space and resources and the nutrient derivation and plant tissue colonization efficiency of the competitors, with weaker endophytic competitors being deprived of specific nutrients and resources [53]. In the present study, there were no visible in vitro antagonistic interactions between *P. polymyxa* (EORB-2) and *F. oxysporum* (EOLF-5) (Figure 6), suggesting a possible mutual or related spatial habitation. In contrast, *P. polymyxa* has a strong antagonist relationship with many pathogenic *Fusaria* sp. [54,55].

## 4. Materials and Methods

### 4.1. Plant Sample Collection

Healthy and disease-free leaves and roots of *E. obtusifolius* plants were harvested from the Pietermaritzburg Campus Botanical Garden at the University of KwaZulu-Natal (latitude 29.625073; longitude 30.403557). The plant material was transferred to the laboratory in sterile biosafety plastic bags filled with ice.

### 4.2. Surface Sterilization and Isolation of Endophytes

The plant material was surface-sterilized following a modified procedure by [23]. The detached infection-free leaves and roots were cleaned under running tap water for 30 min to remove epiphytic microorganisms, dust, and other adhering soil particles. This was followed by washing with Tween 20 detergent (3 drops) for 1 min. Thereafter, the plant material was dipped in 0.1% carbendazim solution with constant agitation for 20 min and then washed four times with sterile distilled water. The root and leaf explants were separately immersed in 70% ethanol for 60 s, followed by a treatment in 2% sodium hypochloride for 60 s, then treated with 70% ethanol for 30 s and finally washed five times with sterile distilled water to remove all traces of the sterilizing agent from the explants. The explants were dried on sterile paper towels and dissected into 2 cm pieces.

Five pieces of the sterilized leaf and root explants were imprinted (pressed) onto freshly prepared Oxoid™ NA and PDA to ascertain the effectiveness of this sterilization protocol. In addition, aliquots (30 µL) of the sterile distilled water that was used in the final rinse of the explants were also plated onto the culture media. The success of the surface sterilization method was confirmed by the absence of microbial growth on the culture media.

The root and leaf explants were placed on agar plates to isolate the endophytes. Bacterial endophytes were isolated from the internal tissues of the sterilized root and leaf explants on freshly prepared NA [56] and incubated at 30 ± 2 °C for 3 days. Fungal endophytes were isolated from the explants on plates containing freshly prepared PDA amended with streptomycin (50 mg/mL) and incubated at 30 ± 2 °C for 7 days following a modified method described by [57]. After incubation, the endophytic bacterial colonies found adjacent to the explants and endophytic fungi filaments that emerged from the internal tissues were carefully transferred to fresh NA and PDA plates, respectively, and incubated as stated above. Pure cultures and sub-cultures were obtained and stored at 4 °C until further use. All sterilization and inoculation activities were performed aseptically on a laminar flow bench.

### 4.3. Extraction and Quantification of Secondary Metabolites from Endophytes

Microbial secondary metabolites were extracted from the isolated endophytic fungi and bacteria using ethyl acetate following the slightly modified methods described by [58] and [59], respectively. The total phenolic contents of the crude extracts obtained from the endophytes were quantified as described by [60], using gallic acid to prepare a standard curve. The total flavonoid contents of the crude extracts obtained from the endophytes were quantified as described by [60], using catechin to prepare a standard curve. There were three replicates per isolate.

### 4.4. Antioxidant Activity of Crude Extracts from Endophytes

The radical scavenging activities of the endophyte crude extracts were assayed in the DPPH radical scavenging assay using the protocol of [61], with BHT used as a negative control. There were three replicates per isolate.

The endophytic isolates of *E. obtusifolius* with free radical scavenging capabilities (low IC_50_ values) and an appreciable quantity of secondary metabolites were further assessed for their drought stress tolerance and in vitro plant growth-promoting potential.

### 4.5. Screening of Endophytic Isolates for Drought Stress Tolerance

The in vitro drought stress tolerance of the selected endophytic isolates was evaluated following a modified method described by [62]. Briefly, the drought stress stimulator PEG 6000 was added to 50 mL Mueller Hinton Broth (MHB) for bacterial isolates and Yeast Malt Broth (YMB) for fungal isolates at various concentrations (0, 10, 20, 30, and 40% *w/v*). A loopful of each bacterial isolate was inoculated into the PEG-supplemented MHB and incubated on an orbital shaker (180 rpm) for 4 days at 25 ± 2 °C. A single disc of fungal isolates (about 1 cm) was excised from freshly prepared fungal isolates, inoculated in the PEG-amended YMB, and incubated at 25 ± 2 °C on an orbital shaker (180 rpm) for 10 days. After the incubation period, the growth of the microbial isolates at different water-deficit levels was estimated spectrophotometrically at OD 600 nm and compared to 0% PEG cultures. Endophytic isolates with high OD values were considered water stress-tolerant species. There were three replicates per treatment.

### 4.6. Evaluation of Plant Growth-Promoting Characteristics of Selected Endophytic Isolates

The results were qualitatively assessed and classified as having strong activity (+++), medium activity (++), low activity (+), or no activity (-). All tests were carried out in triplicate.

#### 4.6.1. Ammonia Production

The production of ammonia by selected *E. obtusifolius* endophytic isolates was investigated using a modified protocol of [23], with Nessler’s reagent in peptone broth. The concentration of ammonia was evaluated using the standard curve (y = 1.488x + 0.022) generated from the standard (ammonium sulfate), and the amount of (NH_4_)_2_SO_4_ was expressed in mM.

#### 4.6.2. Siderophore Production

Siderophore production was qualitatively investigated for the bacterial and fungal endophytic isolates using a modified method of [63] with CAS agar. CAS-blue agar was prepared by dissolving 60.5 mg CAS in 50 mL distilled water, which was added to 10 mL iron (III) solution (1 mM FeCl_3_. 6H_2_O in 10 mM HCl). The mixture was gently mixed with 72.9 mg hexadecyltrimethylammonium bromide dissolved in 40 mL distilled water. The resulting dark blue mixture was then autoclaved at 121 °C for 15 min. Simultaneously, a mixture of 30.24 g piperazine-N, N’-bis (2-ethanesulfonic acid) (PIPES), 15 g agar, 900 mL distilled water, and 50% (*w/w*) NaOH, where the pKa was adjusted to 6.8 with PIPES, was also autoclaved. Finally, the two mixtures were combined and gently agitated to avoid foaming, and thereafter, poured aseptically into plates. Upon solidification, freshly prepared selected bacterial cultures and 1 cm discs of fungal isolates excised from the growing hyphal tips were spot-inoculated in their respective plates and incubated for 7 days at 25 ± 2 °C. The appearance of a yellow/orange or purple halo around the microbial colonies was regarded as a positive result for siderophore production.

#### 4.6.3. HCN Production

The ability of the selected endophytic bacteria and fungi to synthesize hydrogen cyanide was evaluated based on the method of [64]. Each bacterial culture was streaked on Petri dishes containing Luria Bertani agar supplemented with 4.4 g/L of glycine. Similarly, for each of the fungal isolates, mycelial discs were aseptically placed on plates containing PDA augmented with 4.4 g/L of glycine.

#### 4.6.4. Phosphate Solubilization Activity

The phosphate solubilization capability of the selected endophytic bacteria and fungi was measured in triplicate using the method of [65] with Pikovskaya’s (PVK) agar. Inoculated plates were incubated at 27 ± 2 °C for 7 days and closely monitored for the development of clear zones around the bacterial and fungal colonies. The phosphate solubilizing index (PSI) was calculated using the formula of [66].

PSI = Colony diameter + Clear zone diameter/Colony diameter

#### 4.6.5. IAA Production

The modified method of [67] was used to assay the IAA production of the selected bacterial and fungal endophytes.

#### 4.6.6. ACCD Activity

The selected bacterial and fungal endophytic isolates were evaluated for their ability to use ACC as their exclusive source of nitrogen using a modified protocol of [68] using DF minimal salts agar augmented with 3 mM ACC.

### 4.7. Molecular Identification of Endophytes

The two dominant endophytic species from the isolates that had promising in vitro plant growth-promoting properties and drought stress tolerance were identified using molecular tools. The fungus (EOLF5) was grown in PDA media at 27 ± 2 °C for 7 days, after which the fungal mycelia was carefully scraped out and suspended in autoclaved distilled water under sterile conditions. DNA extraction and purification were performed using a Quick-DNA Fungal/Bacterial Kit (Zymo Research, India) following the manufacturer’s instructions. The universal ITS regions (partial sequences of the 18S and 28S recombinant deoxyribose nucleic acid (rRNA) and complete sequences of 5.8S rDNA, ITS1, and ITS2) genes of the fungus were amplified using polymerase chain reaction (PCR) and the ITS primers ITS1-5′-TCC GTA GGT GAA CCT GCG G-3 (forward primer) and ITS4-5′-TCC TCC GCTTAT TGA TAT GC-3′ (reverse primer). Each reaction mixture contained 1 µL extracted DNA in 20 µL PCR reaction mixture. PCR was performed using a thermocycler under the following conditions: minimum initial denaturation at 94 °C, 35 cycles of 30 s denaturation at 94 °C, 30 s minimum annealing at 50 °C and 1 min extension at 68 °C, and 5 min final extension at 68 °C.

The DNA of the bacteria (EORB2) was extracted and purified using the same method as for the fungal species. The PCR composition was the same as mentioned above except that the primers were 907-R and 1492-R. PCR was performed using a thermocycler with the following conditions: 5 min initial denaturation at 95 °C, 30 cycles of 1 min denaturation at 94 °C, 1 min annealing at 55 °C and 1.5 min extension at 72 °C, and 10 min final extension at 72 °C.

The amplified products were electrophoresed in 1% w/v agarose gel electrophoresis with (CSL-AG500, Cleaver Scientific Ltd., Rugby, UK) stained with EZ-vision^®^ Blue light DNA Dye. The cleaned products were injected into an Applied Biosystems ABI 3500XL Genetic Analyzer or Applied Biosystems ABI 3730XL Genetic Analyzer with a 50 cm array, using POP7. The sequence chromatogram analysis was performed using Finch TV analysis software and Bio Edit Sequence Alignment Editor v.7.0. The amplified sequence was deposited in the National Centre for Biotechnology Information (NCBI) database (http://www.ncbi.nlm.gov/BLAST, accessed on 23 November 2022). The sequences thus obtained were submitted to GenBank for their accession numbers.

### 4.8. Endophyte Antagonistic Check Using Dual Culture Method

The two endophytes (EORB-2 and EOLF-5) that exhibited the best in vitro plant growth-promoting potential and drought stress tolerance were evaluated for any possible incompatible growth pattern against each other on PDA plates using the dual antagonistic culture method described by [69]. A 5 mm disc of the fungal isolate was aseptically laid in the middle of the PDA plate, and the bacterial isolate was inoculated at an equal distance from the plate periphery. The organisms were incubated for 7 days at 27 ± 2 °C and observed for any inhibition zone. Compatibility between the two organisms was confirmed by the absence of an inhibition zone, while the establishment of an inhibition zone indicated antagonism between the species. The experiment was conducted in triplicate, and the control plates were inoculated with either of the organisms.

### 4.9. Statistical Analysis

Numerical data obtained from the different assays in this study were analyzed with one-way analysis of variance (ANOVA) using GraphPad Prism 7 (GraphPad Software, Inc., San Diego, CA, USA), and the results are expressed as the mean ± standard error of the means of triplicates. The significance of the means was calculated using Duncan’s multiple range test with *p* values < 0.05.

## 5. Conclusions

This study revealed that *E. obtusifolius*, naturally found in semi-arid areas, hosts a diverse group of fungal and bacterial endophytes. A total of 26 endophytes (fourteen bacteria and twelve fungi) were isolated from the roots and leaves of *E. obtusifolius*. These endophytic species displayed varying in vitro plant growth-promoting and drought stress tolerance capacities. The two most promising water stress-tolerant and plant growth-enhancing endophytic species, namely *P. polymyxa* and *F. oxysporum*, were identified using molecular tools. These two species did not display any form of hostility in the in vitro dual culture experiment. Thus, the data from this study indicate that the inoculation (individually or in combination) of *P. polymyxa* and *F. oxysporum* into plants could potentially promote plant growth and enhance the host’s tolerance to water stress. The characterization and identification *P. polymyxa* and *F. oxysporum* from *E. obtusifolius* elucidated the nature of endophytes residing in the endosphere of medicinal plants and suggests their possible potential as efficient environmentally friendly bio-inoculants for the sustainable cultivation of indigenous plants.

## Figures and Tables

**Table 1:** Quantities of total phenolics, flavonoids, and DPPH IC_50_ values of the crude extracts obtained from the endophytic species isolated from *E. obtusifolius*.

Endophyte Isolates	Phenolics (mg GAE/mg DW of Extracts)	Flavonoids (mg CE/mg DW of Extracts)	% DPPH Radical Scavenging Abilities at 100 µg/mL	DPPH IC_50_ (µg/mL)
EORB-1	4.00 ± 0.02 ^c^	27.66 ± 0.72 ^de^	75.52 ± 1.13 ^de^	48.95 ± 1.09 ^hij^
EORB-2	4.22 ± 0.05 ^c^	32.26 ± 1.10 ^cd^	81.90 ± 0.79 ^bc^	35.68 ± 0.87 ^ij^
EORB-3	-	16.582 ± 0.60 ^ghijk^	10.00 ± 1.08 ^o^	707.48 ± 10.89 ^b^
EORB-4	-	17.21 ± 1.27 ^ghijk^	20.86 ± 2.94 ^m^	256.26 ± 59.23 ^cd^
EORB-5	-	18.04 ± 0.75 ^ghij^	25.86 ± 2.88 ^kl^	195.03 ± 18.34 ^def^
EORB-6	-	16.08 ± 0.75 ^ghijk^	20.86 ± 1.58 ^m^	245.47 ± 24.07 ^cde^
EOLB-1	1.55 0.08 ^g^	19.30 ± 0.21 ^ghi^	68.27 ± 1.77 f^g^	70.76 ± 2.00 ^ghij^
EOLB-2	0.28 ± 0.02 ^k^	14.28 ± 1.46 ^hijklm^	15.49 ± 0.66 ^n^	245.30 ± 2.79 ^cde^
EOLB-3	0.27 ± 0.23 ^k^	10.73 1.00 ^klmno^	67.37 ± 0.66 ^gh^	66.22 ± 1.23 ^ghij^
EOLB-4	2.00 ± 0.05 ^f^	56. 918 ± 6.90 ^b^	80.30 ± 0.84 ^cd^	32.37 ± 0.84 ^ij^
EOLB-5	-	7.60 ± 0.72 ^no^	12.18 ± 1.31 ^no^	352.41 ± 20.74 ^c^
EOLB-6	-	19.72 ± 0.21 ^ghi^	24.81 ± 1.08 l^m^	215.36 ± 14.17 ^def^
EOLB-7	-	12.611 ± 1.90 ^jklmn^	35.94 ± 0.94 ^j^	136.79 ± 7.17 ^j^
EOLB-9	-	11.57 ± 1.30 ^jklmn^	41.20 ± 0.40 ^i^	118.19 ± 2.65 ^i^
EORF-1	6.74 ± 0.30 ^b^	69.04 ± 2.83 ^a^	90.38 ± 0.40 ^a^	23.43 ± 0.18 ^ij^
EORF-3	3.70 ± 0.05 ^d^	38.00 ± 6.88 ^c^	23.76 ± 4.06 ^lm^	222.08 ± 42.44 ^def^
EORF-4	-	13.45 ± 1.50 ^ijklmn^	23.16 ± 1.59 ^lm^	221.44 ± 22.07 ^def^
EORF-5	2.80 ± 0.02 ^e^	16.60 ± 0.42 ^ghijk^	66.62 ± 2.77 ^gh^	62.57 ± 1.65 ^hij^
EORF-6	-	16.60 1.80 ^ghijk^	21.05 ± 1.30 l^m^	248.67 ± 19.55 ^cd^
EORF-8	2.12 ± 0.02 ^f^	26.82 ± 2.54 ^def^	64.36 ± 1.45 ^gh^	64.63 ± 2.067 ^ghij^
EORF-9	0.62 ± 0.02 ^j^	17.63 ± 0.63 ^ghij^	41.05 ± 3.26 ^i^	147.44 ± 10.74 ^defgh^
EOLF-1	1.35 ± 0.07 ^h^	21.60 ± 4.00 ^efg^	63.10 ± 1.24 ^h^	57.31 ± 1.25 ^ij^
EOLF-2	-	8.43 ± 0.60 ^mno^	29.83 ± 1.21 ^k^	172.12 ± 6.08 ^defg^
EOLF-3	1.00 ± 0.04 ^h^	20.76 ± 0.00 ^fgh^	72.24 ± 1.64 ^ef^	47.77 ± 0.68 ^hij^
EOLF-4	-	9.69 ± 0.21 ^lmno^	22.93 ± 1.08 ^lm^	224.78 ± 12.34 ^def^
EOLF-5	9.80 ± 0.03 ^a^	15.33 ± 0.80 ^ghijkl^	86.44 ± 1.88 ^ab^	26. 67 ± 0.889 ^j^
BHT	-	-	86.21 ± 1.53 ^ab^	42.98 ± 3.86 ^hij^

Data represent mean values ± standard error of means (n = 3). Columns with similar letter(s) show non-significant results (*p* > 0.05), and columns with different letter(s) show significant results (*p* < 0.05). - = not detected.

**Table 2:** Qualitative plant growth-promoting traits of endophytic strains isolated from *Endostemon obtusifolius*.

Traits	Endophyte Isolates										
	EORB-1	EORB-2	EOLB-1	EOLB-3	EOLB-4	EORF-1	EORF-5	EORF-8	EOLF-1	EOLF-3	EOLF-5
Ammonia production	+	+++	+++	+	+++	+++	++	+	++	++	+++
Siderophore production	++	+++	+++	+	+	++	++	+	+++	++	+++
Hydrogen cyanide production	+	+++	+	-	+	+	+	+	++	++	++
Phosphate solubilization	+	+	+	-	+	+	+	+	+	+	+
IAA production	+	+	+	-	+	+	-	-	-	+	+
ACC deaminase activity	+	+	+	+	+	-	-	-	-	+	+

+++ Strong activity; ++ medium activity; + low activity; - no activity.

**Table 3:** Molecular identification of two drought-resistant and plant growth-promoting endophytes isolated from *Endostemon obtusifolius*.

Isolate Code	Most Closely Related Homologue Sequence (Accession Number)	Sequence Identity (%)	GeneBank Accession Number
EORB-2	*Paenibacillus polymyxa* (MT163461.1)	100.00	OL619995
EOLF-5	*Fusarium oxysporum* (MT560381.1)	99.60	MZ598577

## Data Availability

All data generated and analyzed for this study is included in the published article.
